# Effectiveness of Optional Videoconferencing-Based Treatment of Alcohol Use Disorders: Randomized Controlled Trial

**DOI:** 10.2196/mental.6713

**Published:** 2017-09-29

**Authors:** Kristine Tarp, Anders Bo Bojesen, Anna Mejldal, Anette Søgaard Nielsen

**Affiliations:** ^1^ Unit of Clinical Alcohol Research Department of Clinical Research University of Southern Denmark Odense C Denmark; ^2^ Centre for Telepsychiatry Department of Psychiatry Region of Southern Denmark Odense C Denmark

**Keywords:** treatment of alcohol use disorders, alcoholism, videoconferencing, effectiveness, adherence, patient compliance, premature dropout, patient dropouts

## Abstract

**Background:**

Treatment of alcohol use disorders (AUDs) is characterized by an adherence rate below 50%. Clinical research has found that patient adherence enhances treatment effect; hence, health authorities, clinicians, and researchers strive to explore initiatives contributing to patients receiving treatment. Concurrently, videoconferencing-based treatment is gaining ground within other addiction and psychiatric areas.

**Objective:**

The aim of this study was to test whether optional videoconferencing increases adherence to and effectiveness of AUD treatment in a randomized controlled trial (RCT). We hypothesized that the intervention would decrease premature dropout (the primary outcome), as well as increase successful treatment termination, treatment duration, and treatment outcome (secondary outcomes).

**Methods:**

We conducted this study in the public outpatient alcohol clinic in Odense, Denmark, between September 2012 and April 2013. It was an RCT with 2 groups: treatment as usual (TAU) and treatment as usual with add-on intervention (TAU+I). The TAU+I group had the option, from session to session, to choose to receive treatment as usual via videoconferencing. Data consisted of self-reported responses to the European version of the Addiction Severity Index (EuropASI). We collected data at baseline, at follow-up at 3, 6, and 12 months, and at discharge.

**Results:**

Among consecutive patients attending the clinic, 128 met the inclusion criteria, and 71 of them were included at baseline. For the primary outcome, after 180 days, 2 of 32 patients (6%) in the TAU+I group and 12 of 39 patients (31%) in the TAU group had dropped out prematurely. The difference is significant (P=.008). After 365 days, 8 patients (25%) in the TAU+I group and 17 patients (44%) in the TAU group had dropped out prematurely. The difference is significant (P=.02). For the secondary outcomes, significantly more patients in the TAU+I group were still attending treatment after 1 year (P=.03). We found no significant differences between the 2 groups with regard to successful treatment termination and treatment outcome.

**Conclusions:**

The results indicate that offering patients optional videoconferencing may prevent premature dropouts from treatment and prolong treatment courses. However, the small sample size precludes conclusions regarding the effect of the intervention, which was not detectable in the patients’ use of alcohol and severity of problems.

**Trial Registration:**

The Regional Health Research Ethics Committee System in Denmark: S-20110052; https://komite.regionsyddanmark.dk/wm258128 (Archived by WebCite at http://www.webcitation.org/6tTL3CO6u)

## Introduction

Treatment of alcohol use disorders (AUDs) is characterized by an adherence rate below 50% [1,2], with 50% of the patients dropping out within the first month of treatment [3,4]. Clinical research has found that the largest alcohol behavior change occurs at the beginning of a treatment course [5] and that patient adherence enhances treatment effect [6]. Hence, health authorities, clinicians, and researchers strive to explore initiatives contributing to patients receiving the planned amount of treatment. Even though research on the optimal duration of treatment is sparse, to our knowledge, no studies found evidence for an increased effect of longer treatment courses compared with shorter courses, such as 3 and 6 months [7-9]. The Danish clinical guideline for the treatment of alcohol dependence [10] recommends a 3-month course of therapy with the possibility of extension. However, most treatment institutions in Denmark have, so far, recommended 6 months of treatment in general or even longer, and this is still often the case.

Videoconferencing-based treatment, either alone or combined with face-to-face treatment such as blended care, has shown great potential for enhancing treatment and recovery within substance use and psychiatric areas, as it decreases barriers of time and distance [11,12]. The field of videoconferencing-based treatment of AUDs is relatively new, and the few existing studies were predominantly small pilot and feasibility studies, which found high levels of patient satisfaction and acceptance [13-19]. Furthermore, they found that videoconferencing may offer the potential to meet some of the challenges AUD treatment is facing regarding barriers [15,16], especially for patients who live at a considerable distance from the clinics [17-19] or have other psychiatric diagnoses [14]. Moreover, these earlier studies found videoconferencing-based assessment and treatment to be highly credible [13,14] and even as effective as face-to-face treatment, with similar relapse and attrition rates [13].

In supplement to the existing studies on videoconferencing-based treatment of AUDs, we have conducted a small randomized controlled trial (RCT) in a real-life setting. The purpose was to examine the effectiveness of optional videoconferencing-based AUD treatment on a laptop with wireless Internet and a videoconferencing client. To our knowledge, this is the first study where AUD patients could opt in on videoconferencing for as many sessions as they chose. However, studies regarding Web-based blended therapy for psychiatric disorders have, for example, examined designs with optional modules [20], with the opportunity to step up treatment if the patient felt it was necessary [21], and using a personal blend [22], enhancing patients’ self-management [12]. Similarly, a qualitative study nested within the RCT found that patients being offered videoconferencing may have experienced it as a means to enhance their autonomy and empowerment, with the ability to choose freely between the two formats having a positive impact on the treatment course [23]. Also, a mixed methods study linked to the RCT found that patients felt more satisfied with the treatment and prolonged their treatment courses when they had the opportunity to receive sessions via videoconferencing [24]. Therefore, it seems highly relevant to examine whether the opportunity of receiving all or some of the treatment course by means of videoconferencing can increase adherence to, and thereby the effectiveness of, AUD treatment.

### Aim

The aim of this study was to test whether optional videoconferencing increases adherence to and effectiveness of AUD treatment in an RCT.

### Hypotheses

We hypothesized that the intervention would decrease the number of patients who dropped out prematurely. We tested this by measuring premature dropout at 6-month follow-up (the primary outcome). Additionally, we hypothesized that the intervention would increase the number of patients terminating their treatment course successfully, increase the proportion of patients still attending a treatment course after 6 months from 45% to 70%, and increase treatment outcome. We tested this by measuring successful treatment termination, treatment duration, and the difference in alcohol characteristics from baseline to 1 year into the treatment course (secondary outcomes).

## Methods

### Design

The study was an RCT with 2 groups: treatment as usual (TAU) and treatment as usual with add-on intervention (TAU+I).

### Sample

All consecutive patients who attended the public outpatient alcohol clinic in Odense, Denmark, between September 2012 and April 2013 were eligible to participate in the study. Inclusion criteria were age 18 years or older, harmful alcohol use or alcohol dependence syndrome according to the *International Classification of Diseases, Tenth Revision* (*ICD-10*), and written informed consent. Exclusion criteria were dementia, psychoses, and lack of sufficient Danish language skills. We registered the study with The Regional Committees on Health Research Ethics for Southern Denmark (S-20110052; Multimedia Appendix 1 [25]).

### Setting

In the outpatient clinic, an interdisciplinary team of social workers, nurses, and psychiatrists conducts the AUD treatment, based on clinical guidelines [26]. The treatment is free of charge and based on face-to-face therapy sessions and pharmacology, if needed [27]. At the beginning of the treatment course, the patients are offered detoxification and motivational interviewing [28]. When they are free of withdrawal symptoms and if they decide to attend a psychosocial treatment course, the patients undergo an assessment interview using the European version of the Addiction Severity Index (EuropASI) [29,30]. Based on an algorithm using the results of the assessment interview, consultant psychiatrists refer the patients to individual psychosocial treatment. This may consist of cognitive behavioral therapy, supportive consultations, family therapy, or contract treatment [31]; as such, there is no difference in the effect of each offer. Treatment is conducted by well-trained and closely supervised nurses and social workers. The length of each treatment course is individually planned. The duration of a typical successful treatment course is about 7 months. One treatment session lasts between 30 and 60 minutes. Session frequency is 1 to 3 times a week at the beginning of the course and 1 session every other week later in the course [32].

### Recruitment and Randomization

**Figure 1:**
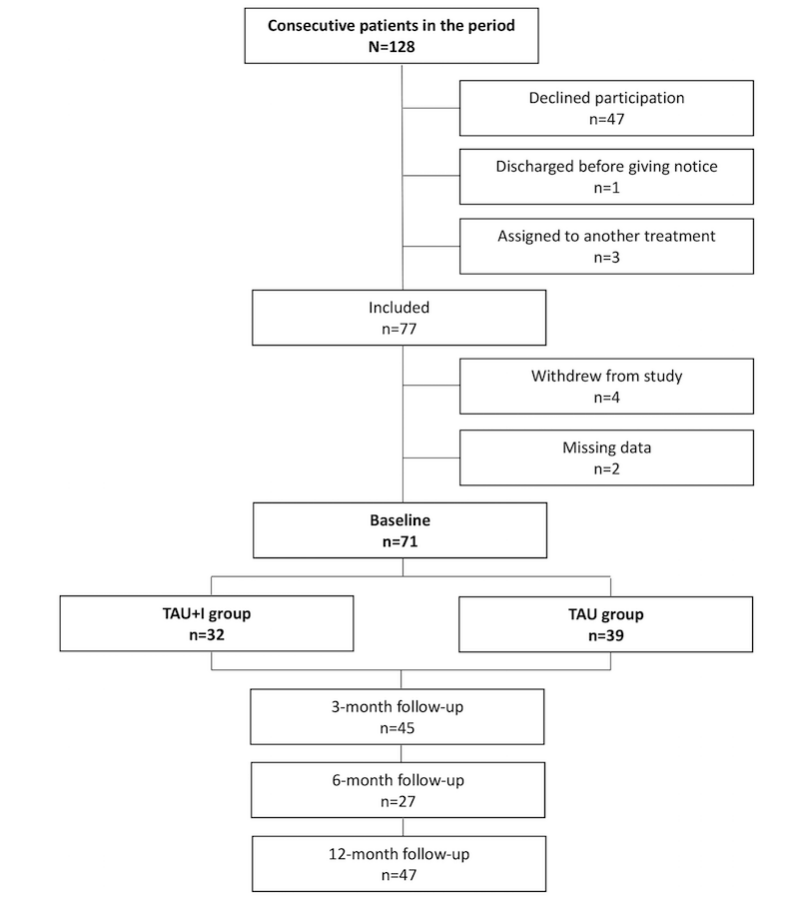
Flowchart of the recruitment process. TAU: treatment as usual; TAU+I: treatment as usual with add-on intervention.

We recruited patients during the assessment interview and systematically offered participation to consecutive patients who decided to attend a psychosocial treatment course and met the inclusion criteria. We included patients who decided to participate in the study and randomly assigned them during the assessment interview; we were aiming at recruiting 140 patients, with 70 in each group. Randomization was carried out by the administrative staff, who were not affiliated with the study. The patients drew a nontransparent envelope packed by an independent person. The envelope contained information about their group placements, either the TAU group or the TAU+I group, with their entailments. Figure 1 shows the recruitment and randomization process.

#### Treatment as Usual

The TAU group received treatment as usual face-to-face at the clinic. Treatment was conducted as described in the Setting section above, according to clinical guidelines.

#### Treatment as Usual With Add-On Intervention

The TAU+I group also received treatment as usual. In addition, they were offered optional videoconferencing and were, from session to session, able to choose to receive treatment as usual via videoconferencing. Hence, the offer of receiving treatment by optional videoconferencing was the intervention in this study. We chose this approach, of patients opting in on videoconferencing as opposed to having all patients receive treatment via videoconferencing, in order to offer the patients the opportunity to make the choices. Each patient in the TAU+I group was equipped with a laptop with wireless Internet and a Cisco TelePresence videoconferencing client (Cisco Systems, Inc, San Jose, CA, USA). Prior to scheduling the first treatment session, we instructed the patients in the use of the equipment and conducted a test to ensure that the equipment was fully functional. Before each therapy session, the patients had the choice between treatment as usual at the clinic and treatment as usual via videoconferencing from any location. If the patients opted for a session via videoconferencing, they just needed to turn on the equipment. If the therapist went to fetch the patients from the waiting room, only to discover them absent, the therapist would then call them via videoconferencing.

### Measures

The Addiction Severity Index (ASI) was developed especially for assessment, treatment, and research in addiction [29,33]. Studies have demonstrated the ASI to be a valid instrument [34,35]. The EuropASI [30] provides sociodemographics, alcohol measures, and composite scores for 9 potential problem areas in the patients’ life circumstances. The composite scores reflect the severity of the problems during the last month preceding the assessment interview. The composite scores range from 0 to 1; the higher the score, the greater the severity [29].

Baseline measures were EuropASI sociodemographics about age, sex, higher/continuing education, employment, and cohabitation; EuropASI alcohol measures regarding dependence, age at onset of excessive use of alcohol (≥5 units a day, at least 3 days a week during the last 30 days), years of excessive alcohol use in life, days of alcohol use in the past month, and days of excessive alcohol use in the past month; and EuropASI composite scores regarding alcohol use, drug use, economic status, employment, legal status, family status, social status, medical status, and psychiatric status. The composite scores were computed according to the EuropASI [30].

The primary outcome was premature dropout at the 6-month follow-up. Secondary outcomes were successful treatment termination, treatment duration (measured by the number of days in treatment), and alcohol characteristics, consisting of 12-month follow-up alcohol use and severity (measured by the number of days of alcohol use and excessive alcohol use in the past month preceding the interview), and composite scores regarding alcohol use, employment, legal status, family status, medical status, and psychiatric status.

### Data Collection

Baseline data were collected by the therapists at the assessment interview at treatment start prior to the randomization. Follow-up data were collected by the therapists as part of the routine treatment course as long as the patients attended treatment, at the status sessions at 3, 6, and 12 months, and at discharge. We collected additional 1-year follow-up data. To collect the data, we used letters, telephone calls, and personal contacts to secure the highest possible participation rate [36].

In addition, we collected data on the actual use of videoconferencing, and from questionnaires on nonparticipation and satisfaction, and semistructured interviews with participants in the TAU+I group, therapists, and collaborators. We used these additional data for separate analyses of nonparticipation, satisfaction, patient perspectives, and therapist perspectives on the use of videoconferencing.

### Statistics

We conducted the analyses in this study using SAS Enterprise Guide 7.1 (SAS Institute Inc) and Stata v14 (StataCorp LLC).

The power was calculated in Stata. The primary outcome was the number of patients still attending treatment 6 months after the assessment interview. The calculation was based on numbers from the clinic in 2010 showing that the proportion of patients still attending treatment after 7 months was 45%. We expected the intervention to increase the proportion of patients still attending a treatment course after 6 months from 45% to 70%. For a significance level of 5% and a power of 80%, 140 patients should be included, with 70 in each group.

Baseline variables, sex, higher/continuing education, employment, cohabitation, and dependence were categorical; hence, we used Pearson chi-square tests for relationships between variables. The variables higher/continuing education and dependence had an expected frequency of 5 or less in one cell; hence, we used Fisher exact test. The rest of the variables were continuous; hence, we used the Shapiro-Wilk *W* test for normal data to check for normally distributed data. None of the continuous variables were normally distributed; hence, we used 2-sample Wilcoxon rank sum (Mann-Whitney) tests to compare the means of not normally distributed interval-dependent variables for 2 independent groups.

We tested the primary outcome, premature dropout at 6-month follow-up, by means of a Kaplan-Meier survival analysis using Wilcoxon test statistics.

Secondary outcomes were regarding successful treatment termination, treatment duration, and alcohol characteristics. We tested successful treatment termination by means of a logistic regression analysis. We tested treatment duration by means of a Kaplan-Meier survival analysis at 6-month and 12-month follow-ups. We tested differences in alcohol characteristics from baseline to 1-year follow-up using per-protocol analyses; however, due to missing data, we used last observation carried forward. The variables were continuous; hence, we used the Shapiro-Wilk *W* test for normal data to check for normally distributed data. The variable employment was normally distributed; hence, we used a 2-sample *t* test with equal variances to compare means. The rest of the continuous variables were not normally distributed; hence, we used 2-sample Wilcoxon rank sum (Mann-Whitney) tests to compare means. We made no corrections for multiple comparisons.

## Results

### Participants

Our goal was to recruit 140 participants, but we succeeded in recruiting only 71 participants. As the flowchart in Figure 1 shows, 128 consecutive patients entered psychosocial AUD treatment during the period of recruitment. A total of 47 patients declined to participate in the study, 3 patients were assigned to another treatment, and 1 patient was discharged before giving notice. After inclusion, a further 4 patients withdrew from the study, and data were missing for 2. Hence, only 71 patients completed the baseline assessment interview and were randomly assigned: 39 patients to the TAU group and 32 patients to the TAU+I group.

**Table 1:** Baseline sample characteristics, by randomization group (N=71).

Characteristics		TAU^a^ group (n=39)	TAU+I^b^ group (n=32)	*P* value
**EuropASI^c^** **sociodemographics**				
	Age in years, mean (SD)	47.3 (12.4)	46.0 (13.5)	.64
	Sex (female), n (%)	10 (26)	9 28)	.81
	Higher/continuing^d^ education (yes), n (%)	30 (77)	28 (88)	.36
	Employed^e^ (yes), n (%)	20 (51)	11 (34)	.15
	Cohabiting (yes), n (%)	22 (56)	20 (63)	.60
**EuropASI alcohol measures**				
	Alcohol dependence^f^ (yes), n (%)	32 (82)	28 (87)	.74
	Age in years at onset of excessive^g^ use of alcohol, mean (SD)	31.31 (13.72)	32.25 (14.83)	.73
	Years of excessive alcohol use in life, mean (SD)	16.28 (10.51)	13.09 (11.79)	.09
	Days of alcohol use in the past month, mean (SD)	18.44 (10.89)	20.44 (10.37)	.44
	Days of excessive^g^ alcohol use in the past month, mean (SD)	15.54 (11.53)	18.25 (10.24)	.31
**EuropASI composite scores^h^**				
	Alcohol use, mean (SD)	0.68 (0.22)	0.72 (0.19)	.51
	Drug use, mean (SD)^i^	0.02 (0.08)	0.05 (0.12)	.1
	Economic status, mean (SD)	0.54 (0.45)	0.65 (0.45)	.33
	Employment, mean (SD)	0.38 (0.41)	0.44 (0.39)	.54
	Legal status, mean (SD)^j^	0.01 (0.04)	0.04 (0.15)	.24
	Family status, mean (SD)	0.22 (0.27)	0.11 (0.21)	.09
	Social status, mean (SD)^j^	0.08 (0.19)	0.08 (0.19)	.90
	Medical status, mean (SD)	0.29 (0.40)	0.29 (0.39)	.94
	Psychiatric status, mean (SD)	0.20 (0.20)	0.24 (0.26)	.80

^a^TAU: treatment as usual.

^b^TAU+I: treatment as usual with add-on intervention.

^c^EuropASI: European version of the Addiction Severity Index.

^d^Some respondents with continuing education attended high school first; some did not.

^e^Not necessarily full time.

^f^According to the *International Classification of Diseases, Tenth Revision* (*ICD-10*).

^g^≥5 units a day in at least 3 days a week during the last 30 days.

^h^EuropASI composite scores vary from 0 (no problem) to 1 (extreme problem) in the 30 days preceding the interview.

^i^On the basis of 69 observations.

^j^On the basis of 70 observations.

### Baseline

Table 1 shows baseline characteristics of the participants. The 2 groups received the same variation of treatment offers. The average participant was about 47 years old, most were male, and more than half were cohabiting. A majority had higher/continuing education but less than 50% were employed. More than 80% had a diagnosis of alcohol dependence syndrome according to the *ICD-10*. The 2 groups did not deviate from each other according to EuropASI sociodemographics, alcohol measures, and composite scores. It seems that the allocation of patients to the 2 groups resulted in 2 similar groups with regard to sociodemographic and alcohol characteristics. Therefore, we assumed that the randomization was successful.

### Use of Videoconferencing

Records of the use of the intervention showed that 16 of the 32 patients (50%) in the TAU+I group used the laptop with videoconferencing for a total of 60 treatment sessions; however, 37 (62%) of the sessions had technical problems. Mostly, these problems consisted of poor sound quality, which was solved by using telephones for the sound.

### Primary Outcome: Premature Dropout

The termination status of the patients in this study fell into 2 categories. The first category is premature dropout, consisting of patients who did not appear at the discharging session but were expected to return, patients who were discharged after not appearing at the treatment sessions, and patients who were discharged by their own wish (at a time considered by the therapist as being too early).

**Figure 2:**
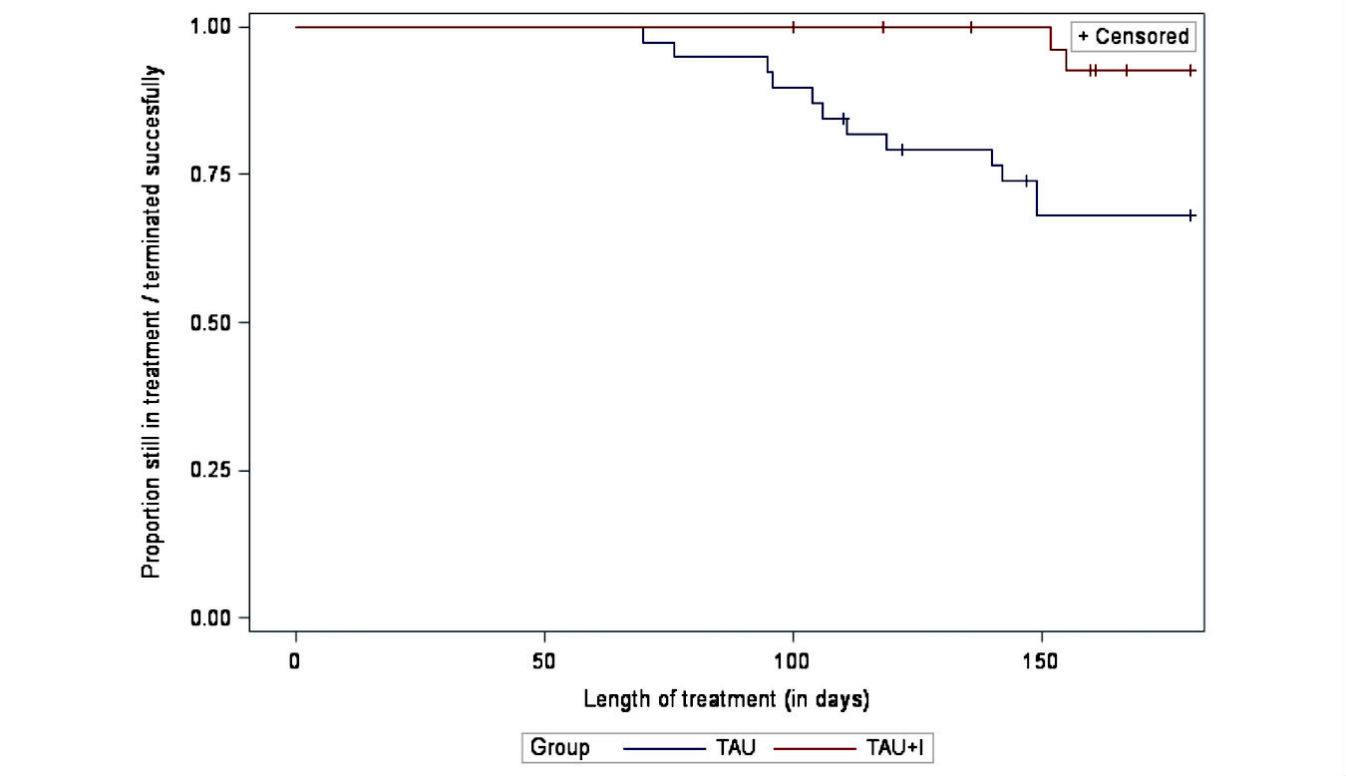
Primary outcome: premature dropout; survival curves (*P*=.008; successful terminations censored), by randomization group (N=71; treatment as usual [TAU] group: n=39; treatment as usual with add-on intervention [TAU+I] group: n=32).

Figure 2 shows premature dropout by the means of a survival analysis. The plot shows the number of days the TAU group and the TAU+I group stayed in treatment or the number who successfully terminated treatment. After 180 days in treatment, 2 of 32 patients (6%) in the TAU+I group and 12 of 39 patients (31%) in the TAU group had dropped out prematurely. The difference is significant (*P*=.008). After 365 days, 8 patients (25%) in the TAU+I group and 17 patients (44%) in the TAU group had dropped out prematurely. The difference is significant (*P*=.02).

### Secondary Outcomes

#### Successful Treatment Termination

The second category of termination status is successful treatment termination, consisting of patients who completed their treatment course as planned or still were in treatment at the discharging session. Upon completion of their treatment course, 21 of 39 patients (54%) in the TAU group and 19 of 32 patients (59%) in the TAU+I group had successfully terminated treatment. The difference is not significant (*P*=.64). The crude odds ratio for successful termination is 1.25 (95% CI 0.48-3.25) for the TAU+I group. When adjusted for employment and sex, the odds ratio for successful termination is 1.57 (95% CI 0.57-4.37).

#### Treatment Duration

**Figure 3:**
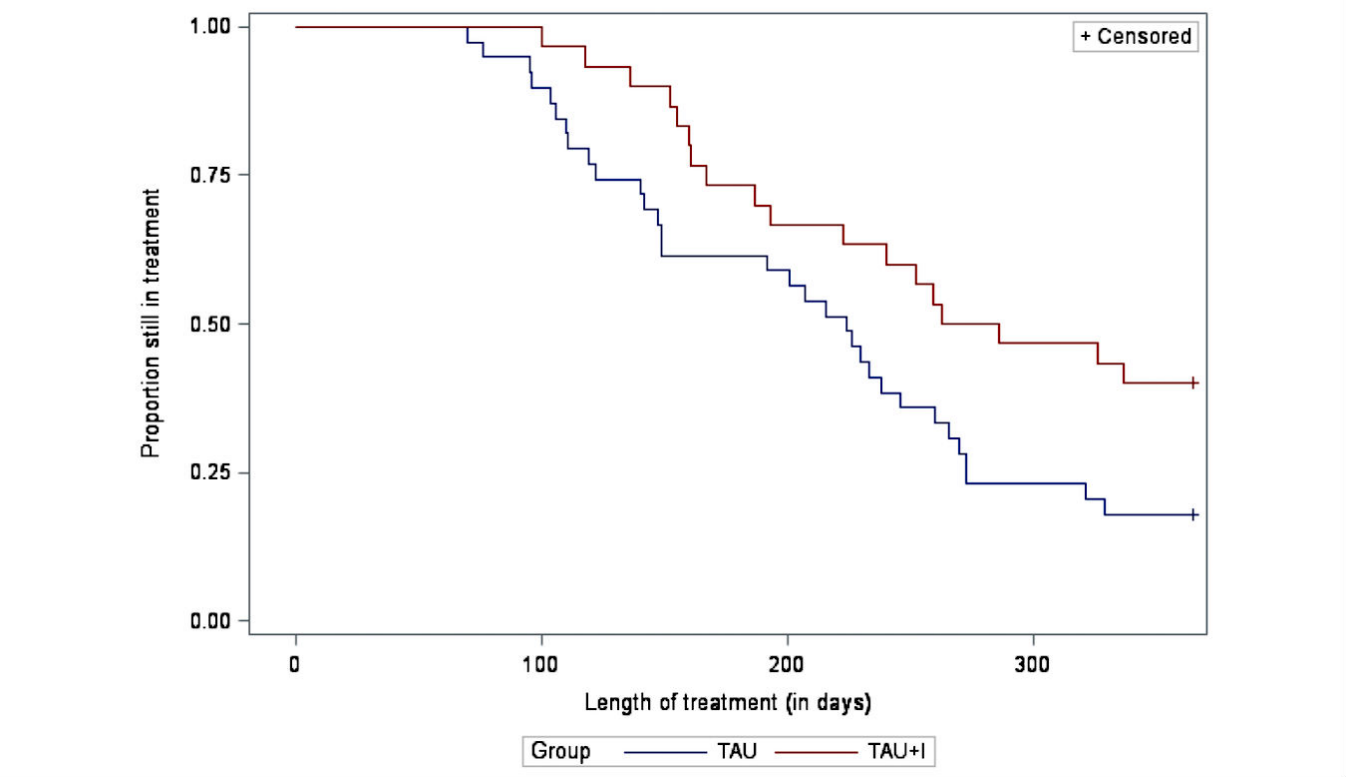
Secondary outcome: treatment duration; survival curves (*P*=.03), by randomization group (N=71; treatment as usual [TAU] group: n=39; treatment as usual with add-on intervention [TAU+I] group: n=32).

Figure 3 shows that after 6 months, 24 of 32 patients (75%) in the TAU+I group and 24 of 39 patients (62%) in the TAU group were still attending treatment. After 1 year, 14 of 32 (44%) patients in the TAU+I group and 7 of 39 (18%) patients in the TAU group were still in treatment. The difference is significant (*P*=.03).

**Table 2:** Changes from baseline to 12-month follow-up, by randomization group (N=71).

Measures		TAU^a^ group (n=39)	TAU+I^b^ group (n=32)	*P* value
**EuropASI^c^** **alcohol measures**				
	Days of alcohol use in the past month prior to interviews, mean (SD)	–12.44 (10.53)	–13.75 (12.40)	.64
	Days of excessive^d^ alcohol use in the past month prior to interviews, mean (SD)	–12.26 (11.29)	–13.47 (11.79)	.68
**EuropASI composite scores^e^**				
	Alcohol use, mean (SD)	–0.48 (0.26)	–0.49 (0.33)	.59
	Employment, mean (SD)	–0.05 (0.33)	–0.05 (0.42)	.97
	Legal status, mean (SD)^f^	–0.01 (0.03)	–0.01 (0.05)	.21
	Family status, mean (SD)	–0.09 (0.25)	–0.02 (0.25)	.52
	Medical status, mean (SD)^g^	0.07 (0.42)	–0.03 (0.21)	.14
	Psychiatric status, mean (SD)	–0.04 (0.24)	0.00 (0.21)	.42

^a^TAU: treatment as usual.

^b^TAU+I: treatment as usual with add-on intervention.

^c^EuropASI: European version of the Addiction Severity Index.

^d^≥5 units a day in at least 3 days a week during the last 30 days.

^e^EuropASI composite scores vary from 0 (no problem) to 1 (extreme problem) in the 30 days preceding the interview.

^f^On the basis of 70 observations.

^g^On the basis of 69 observations.

#### Alcohol Characteristics

Table 2 shows differences from baseline to 1-year follow-up for selected alcohol measures and composite scores. We found no significant differences.

## Discussion

The results of this study indicate that the offer of optional videoconferencing may prevent premature dropout, in which patients attend only the first couple of sessions and then drop out. One reason why patients in the TAU+I group had significantly fewer premature dropouts may have been that they were more satisfied with their treatment course, having been given the opportunity to choose (before each session) whether to receive treatment via videoconferencing [24]. This may have led to increased experiences of flexibility and autonomy, making the patients feel more empowered [23], which, in turn, may have prevented dropouts. Similar notions were reported in a study on Web-based blended care therapy, where patients had positive perceptions of the Web-based sessions, especially regarding enhancing their self-management [12]. Hence, videoconferencing may have encouraged adherence to treatment and prevented premature dropouts. This is especially interesting, since previous research found that the largest alcohol behavior change occurred at the beginning of a treatment course [5]. Previous research also found that patient groups with a general lack of dependability (eg, no job stability, psychiatric illness, and prior discharges from hospitals) tend to drop out prematurely. However, patients functioning too poorly or too well may both equally increase dropout rates. This study investigated videoconferencing as a means to reduce premature dropout, but videoconferencing is, of course, only one of many initiatives that may be used. Other useful examples include reduction of waiting time at the beginning of the treatment course [9,26], treatment matching [6,37], explanation of the anticipated treatment [9], use of clinical guidelines [26,38-41], a less focal and talkative therapist at the beginning of the treatment course [9], engagement of relatives in the treatment process [9], aggressive pursuit [9], use of attendance contracting and prompting [42], and contact with no-shows [43].

The Danish clinical guideline for treatment of alcohol dependence [10] recommends treatment courses of 3 months’ duration with the possibility of extension; hence, we chose to measure 6 and 12 months after treatment start, allowing most patients to have completed the treatment course. In 2010, 45% of the patients attending the clinic were still in treatment after 6 months. We expected this intervention to increase this proportion from 45% to 70%. After 6 months, 75% of the patients in the TAU+I group were still attending treatment, which exceeds the expected increase. However, also after 6 months, 62% of the TAU group were still attending treatment, which well exceeds the proportion from 2010. Nonetheless, it may be an ongoing issue to offer treatment courses that comply with cost effectiveness; hence, in a review from 2014, Littrell [9] explored outcome findings regarding length of treatment. Several reviews have correlated treatment duration with outcome and found that patients who remain in treatment longer have better outcomes. Hence, it seems that patients who drop out have poorer outcomes. However, some of these studies did not consider whether the duration was shorter because of planned termination or premature dropout. Studies where patients were randomly assigned to longer or shorter treatment durations, and studies comparing shorter versus longer treatment programs, have not found any differences in outcomes, except for patients with lower socioeconomic standing [9]. More than 80% of the patients in this study had alcohol dependence syndrome diagnosed according to the *ICD-10*. Evidently, patients with severe drinking problems, and without social support, benefit from treatment [9]; hence, research suggests that patients with moderate or severe levels of alcohol dependence should be referred to and encouraged to attend treatment [44].

Despite significantly fewer premature dropouts in the TAU+I group, it was not possible to detect any significant differences in the effect of the treatment after 1 year with regard to alcohol consumption. There could be several reasons why outcome did not differ between the 2 groups. For instance, patients without the option of videoconferencing, but still motivated to attend face-to-face sessions, may have been more motivated in general and thereby produced better outcomes. In contrast, those who had the option could have been less motivated in general, less willing to appear face-to-face, and more willing to use the videoconferencing option instead. Another reason may have been that the poor technical quality of the equipment the patients were provided with, especially the sound where phones were often used instead, may have caused the patients to need further sessions. This would, unintentionally, have increased treatment duration, as these patients probably did not fully benefit from the videoconferencing treatment sessions due to poor technical quality and hence only maintained their treatment status quo. Here, those who put up with the poor technical quality of the equipment handed to them would probably have been more motivated to change, compared with those who did not. A few previous studies on videoconferencing-based treatment of AUDs have addressed attendance and effect. Frueh et al found that 13 out of 14 patients who completed their study remained abstinent throughout the treatment [13]. Staton-Tindall et al [16] found no significant differences between the intervention group and the comparison group receiving motivational enhancement therapy via videoconferencing. However, sessions 3 to 5 (focusing on change) of the intervention significantly reduced the likelihood of using alcohol by 72% and predicted fewer drinking days, fewer drinks per week, and fewer days experiencing problems with alcohol during the follow-up period; however, both motivational enhancement therapy and videoconferencing were part of the intervention [16].

### Strengths and Limitations

The most important strength of this small RCT is that it was carried out as an effectiveness study in a real-life setting, where consecutive patients seeking ordinary AUD treatment at the outpatient clinic were offered participation in the study. Studies conducted among a treatment-seeking population are unique and rarely seen. If an experimental intervention in a research study is likely to be implemented and upscaled in real-life praxis, it is an advantage that the research has been carried out among alcohol patients attending an operating clinic. Effectiveness studies generate results that can inform clinical practice [45,46], and examination of the intervention’s effectiveness, when implemented within an everyday clinical setting, is an important part of establishing an evidence base for a particular treatment [47].

However, as a consequence, the findings of this study may not be as positive as findings from other studies with other prerequisites. Most of the previous studies on videoconferencing-based AUD treatment were small feasibility studies or randomized studies with paid patient participation. Our sample was, nevertheless, fairly similar to them regarding sociodemographic and alcohol measures [13-15,17]. Since the study is representative, it can be generalized to the extent of treatment-seeking patients with harmful alcohol use or alcohol dependence, at the higher severity end, attending clinics in Denmark and other countries with a similar organization of, and distances between, clinics.

A severe limitation of this study is that we were unable to include the number of patients estimated in the power calculations prior to study start. The relatively low number of participants may have been due to patient rates being lower than expected, compared with the same time period in previous years in the same setting. Also, it may have been due to the participation rate being lower than expected, based on participation rates in other studies conducted in the same setting. In alcohol treatment and research, it is a common challenge to recruit and maintain patients for studies, as well as for treatment [48]. Unwillingness to participate in research studies has been reported as becoming more and more common [36], especially regarding studies performed over the Internet [49]. Thus, more patients than anticipated may simply have declined participation because of the technical element in the study. As a consequence, the small sample size in this study is a limitation for the significance of the results and may, thereby, have consequences for the inferential conclusions that can be drawn from the results.

It is a huge strength that data on premature dropout and treatment termination were available for all but 2 patients; however, it may be a limitation that we have 1-year follow-up data for only 66%. Prior studies have reported follow-up participation rates below 60% with no evidence of bias [36], and the use of last observation carried forward is a conservative approach to secure validity. In comparison, previous studies on videoconferencing-based AUD treatment have reported relatively good session attendance and successful intervention engagement, as well as completion rates similar to face-to-face treatment [13,15,16], completion rates ranging from 50% to almost 100% [14,15,17], and follow-up rates of up to 90% [16,18]. This may be due to the fact that most of the prior studies were small pilot and feasibility studies or RCTs, where participants were even paid to participate. These recruitment processes may have biased participation in the prior studies in a positive direction compared with participation in effectiveness studies like ours, where participants were consecutive patients seeking treatment for alcohol problems in a real-life setting.

It may be a limitation to the study that the results were based on self-reported EuropASI data. Even though general population surveys have found alcohol consumption to be underreported, and the accuracy of an individual’s report may be difficult to determine, the literature suggests that, from a group perspective, self-reports of alcohol use from clinical and nonclinical samples are accurate provided that people are interviewed under the following conditions: alcohol-free when interviewed; given written assurances of confidentiality; interviewed in a setting encouraging honest reporting; asked clearly worded objective questions; and provided with memory aids [50].

Furthermore, it may be a limitation to this study that we analyzed psychosocial treatment as a single treatment approach, even though it consists of 4 different psychosocial treatment forms. Since the offers were equally effective and the 2 groups received the same variation in treatment offers, we did this to limit the different outcome possibilities as opposed to limiting any broad inferences about the effects of offering videoconferencing.

Moreover, it may be both a strength and a limitation to have chosen videoconferencing as an option in order not to force any patient to use it. None of the previous similar studies have used *optional* videoconferencing; however, blended care is commonly used in psychiatric treatment. Here, patients reported advantages such as having met the therapist before or during the treatment course [12], and optional use of videoconferencing, throughout the treatment course, offers similar advantages.

### Conclusion

The aim of this study was to test whether optional videoconferencing increases adherence and effect in AUD treatment. We tested this by examining premature dropout (the primary outcome), as well as successful treatment termination, treatment duration, and alcohol characteristics (secondary outcomes). The results indicate that offering patients optional videoconferencing may prevent premature dropouts from treatment and prolong treatment courses. However, the small sample size precludes conclusions regarding the effect of the intervention, which was not detectable in the patients’ use of alcohol and severity of problems. Even though videoconferencing did not, in this study, seem to lead to a more effective treatment course, it may be a tool to increase adherence. Thus, future research is warranted on how videoconferencing and treatment duration may influence adherence and effect in AUD treatment.

## Appendix

Multimedia Appendix 1 CONSORT-EHEALTH checklist V1.6.1.

## Abbreviations

ASI: Addiction Severity Index
AUD: alcohol use disorder
EuropASI: European version of the Addiction Severity Index
ICD-10: International Classification of Diseases, Tenth Revision
RCT: randomized controlled trial
TAU: treatment as usual
TAU+I: treatment as usual with add-on intervention
